# Copolymer-1 enhances cognitive performance in young adult rats

**DOI:** 10.1371/journal.pone.0192885

**Published:** 2018-03-01

**Authors:** Rossana Nieto-Vera, Nicolas Kahuam-López, Alfredo Meneses, Yolanda Cruz-Martínez, Rosa María Anaya-Jiménez, Gustavo Liy-Salmerón, Horacio Guillermo Carvajal, Maria Teresa Ponce-López, Antonio Ibarra

**Affiliations:** 1 Centro de Investigación en Ciencias de la Salud (CICSA), Facultad de Ciencias de la Salud, Universidad Anáhuac México Campus Norte; Av. Universidad Anáhuac No. 46, Lomas Anáhuac, Huixquilucan, Estado de México, México; 2 Departamento de Farmacobiología, CINVESTAV-IPN, México City, México; University of Queensland, AUSTRALIA

## Abstract

Cognitive impairment is a dysfunction observed as a sequel of various neurodegenerative diseases, as well as a concomitant element in the elderly stages of life. In clinical settings, this malfunction is identified as mild cognitive impairment. Previous studies have suggested that cognitive impairment could be the result of a reduction in the expression of brain-derived neurotrophic factor (BDNF) and/or immune dysfunction. Copolymer-1 (Cop-1) is an FDA-approved synthetic peptide capable of inducing the activation of Th2/3 cells, which are able to release BDNF, as well as to migrate and accumulate in the brain. In this study, we evaluated the effect of Cop-1 immunization on improvement of cognition in adult rats. For this purpose, we performed four experiments. We evaluated the effect of Cop-1 immunization on learning/memory using the Morris water maze for spatial memory and autoshaping for associative memory in 3- or 6-month-old rats. BDNF concentrations at the hippocampus were determined by ELISA. Cop-1 immunization induced a significant improvement of spatial memory and associative memory in 6-month-old rats. Likewise, Cop-1 improved spatial memory and associative memory when animals were immunized at 3 months and evaluated at 6 months old. Additionally, Cop-1 induced a significant increase in BDNF levels at the hippocampus. To our knowledge, the present investigation reports the first instance of Cop-1 treatment enhancing cognitive function in normal young adult rats, suggesting that Cop-1 may be a practical therapeutic strategy potentially useful for age- or disease-related cognitive impairment.

## Introduction

Numerous studies have shown that traumatic brain injury, stroke, or even spinal cord injury induce cognitive impairment (CI) [1–3]. Likewise, it has been reported that 10–20% of humans aged 65 years and older may also present CI, a major public health concern with a rapidly increasing incidence worldwide [4]. In order to address this scenario, clinicians face the challenge of identifying clinically significant cognitive changes for the diagnosis of CI [5]. In this regard, longitudinal studies have demonstrated multiple patterns of cognitive change with variable clinical outcomes, including dementia and mild cognitive impairment (MCI) [6]. Dementia is typically diagnosed when acquired CI has become severe enough to compromise social and/or occupational functions, while MCI is an intermediate stage in the trajectory from normal cognition to dementia [5]. The cognitive changes induced by MCI are generally not severe enough to interfere with daily life or independent function; however, it has been demonstrated that patients with MCI have an increased risk of eventually developing Alzheimer’s or other types of dementia [4,5]. The archetypal symptom reported by patients with MCI is memory loss. Currently, there are no medications approved by the U.S. Food and Drug Administration (FDA) to treat MCI.

Previous studies have suggested that the immune system supports cognitive function, and immune function may therefore have far-reaching implications for cognitive disorders [7]. For instance, in elderly individuals, CI is associated with a significant reduction in the activity of the immune system [8]. In line with this, recent studies have shown that the CI observed in aged mice can be reverted through immunization with neural-derived peptides [7]. By stimulating the immune system, this strategy has demonstrated an improvement in cognitive function. Copolymer-1 (Cop-1), a synthetic peptide consisting of four amino acids (alanine, lysine, glutamic acid, and tyrosine) in a fixed molar ratio [9], has been approved for the treatment of relapsing-remitting multiple sclerosis (MS). Subcutaneous administration of Cop-1 has pleiotropic properties, such as: i) an inhibitory effect on monocyte reactivity, limiting their production of tumor necrosis factor α (TNFα) and interleukin-12 (IL-12), and increasing IL-10 and transforming growth factor β (TGFβ) levels; ii) activation of Th2/3 and regulatory T cells (Treg); iii) migration of Cop-1-specific Th2/3 cells across the blood-brain barrier, which secrete neurotrophins (especially brain derived neurotrophic factor, BDNF) and anti-inflammatory cytokines; iv) elevated proliferation of neural precursor cells (NPC) and their recruitment to the injury site [10]. Due to these beneficial actions, especially the stimulation of BDNF secretion and neurogenesis, COP-1 presents an attractive potential option for improving cognition, and possibly in the treatment of CI [11–13]. Recent studies support this assessment, with administration of this compound resulting in improved cognition in a model of 2-vessel-occlusion cognitive deficit [14].

In the present study, we performed a preliminary evaluation on the ability of Cop-1 to enhance cognitive performance in normal young adult male rats.

## Materials and methods

### Ethics statement

All animals were handled according to NIH guidelines for the management of laboratory animals. All the procedures were performed in accordance to the National Institutes of Health *Guide for the care and use of laboratory animals* and the Mexican Official Norm on the Principles of Laboratory Animal Care (NOM 062-ZOO-1999). The study was approved by the Institutional Animal Care and Use Committee and Institutional Review Board of Universidad Anahuac Mexico Norte (ID: 201102). All experiments were designed and reported according to the ARRIVE guidelines. In order to perform euthanasia, animals were previously anesthetized by intramuscular injection with a mixture of ketamine (50 mg/kg) and xylazine (10 mg/kg).

### Animal care

Animals were matched for age and weight in each experiment and housed in pairs in a light and temperature-controlled room. To minimize stress, animals were handled daily at least once a day during the 7 days prior to any procedure.

Sterile bedding and filtered water was replaced daily. All rats were carefully monitored for evidence of complications. Animals with signs of infections were excluded from the study.

### Study design

In order to evaluate the effect of Cop-1 therapy on cognitive function, we performed four experiments. Each study included 2 groups of male Sprague Dawley rats: group 1) Cop-1 administration (n = 8); group 2) saline solution administration (n = 8). In the first experiment, we explored the effect of Cop-1 on the recovery of spatial memory in adult rats. For this purpose, 6-month-old animals were immunized with Cop-1 and, two weeks after treatment, were evaluated daily for 15 days using the Morris water maze (MWM) behavioral test. In the second experiment, we intended to elucidate whether Cop-1 therapy improves cognitive function when administered during the early stages of life. In this case, young rats (3 months old) were treated with Cop-1. Two weeks after therapy administration, rats were evaluated daily during 15 days using the MWM test. In order to investigate whether the effect of Cop-1 was lasting, the same rats were subsequently evaluated in the same manner 3 months later, at 6 months of age. The third and fourth experiments were designed to evaluate associative memory using the Pavlovian autoshaping test. In the third experiment, 6-month-old rats were treated and evaluated two weeks later. The last experiment consisted of 3-month-old rats treated with Cop-1, which were likewise evaluated 3 months later at 6 months of age. After evaluation of groups 3 and 4, 4 rats were randomly selected from each group to determine BDNF concentrations in the hippocampal area. Determinations were performed using the ELISA technique.

### Cop-1 immunization

Animals were injected with 0.71μg/g of body weight of Cop-1 (Sigma, St. Louis, USA) dissolved in saline solution in a total volume of 150μl. This injection was administered once per week during 3 consecutive weeks. Control animals received the same volume of saline solution. All doses were applied subcutaneously at the interscapular space. The only adverse event observed after administration was erythema at the injection site in 4/70 animals.

### Morris water maze test

The MWM was used to assess spatial learning and memory. In this test, animals are placed in a tub full of water, and required to find their way onto a platform hidden just beneath surface level. The location of the platform can only be encoded relative to distal visual landmarks surrounding the water maze pool, thereby using spatial memory. Stable visual cues can be provided using a curtain around the pool. The swim path of the animal is recorded with a camera attached to the ceiling, which is connected to a computer with a tracking software. Based on the time-tagged XY-coordinates of the rat, the software calculates the latency time to reach the hidden platform, a parameter of spatial learning performance [15]. For this study, rats were placed at different starting positions in a circular pool (diameter 120cm) filled with water (21–22°C, made opaque by adding powdered milk). Rats were trained to find a platform (diameter 10cm), which was submerged 1cm below the water surface and located in the north-east quadrant of the pool, by using distant visual cues. These visual cues were present on the four walls surrounding the pool at a distance of 0.6m. During all trials, an observer was present and always located in the same position behind a curtain surrounding the set-up. We used a reference memory protocol with four-day training, followed by 15 days of individual trials. For the training phase, each rat performed 4 acquisition trials (maximal swimming time 120s; 30s on platform; inter-trial interval 30 minutes) per day during 4 consecutive days. Starting positions were south, north, east, and west. Control and Cop-1 rats used the same starting position for each particular day of evaluation. All trials were recorded, and latency time, defined as the delay in finding the platform, was used as a measure for spatial learning. During the trial phase, the rat performed a single daily trial for 15 days. Rats were allowed to swim freely for 120s, and trials were recorded and analyzed with Smart Video Panlab-Harvard software.

### Autoshaping learning task

In order to evaluate associative memory, the autoshaping test, which has been previously described [16–18] (see also YouTube, phrasing: autoshaping and lever-press or nose-poke), was used. For this test, four experimental chambers were used (Coulbourn Instruments, Lehigh Valley, PA). Each apparatus included a standard attenuation system, and one chamber had the following inner dimensions: 25 cm width, 29 cm in length, and 25cm in height. A retractile and illuminable lever-press operandum was mounted 4 cm above the floor and 10 cm from the right and left walls. A food magazine for rat pellets (see below) was located 5 cm to the right of the lever and 3 cm above the floor. A house light was located in the right top corner, which remained turned on for the duration of the session. Computer software was used for control and recording.

#### Food-magazine training

Each rat was individually placed in an experimental chamber for a habituation period (≈15 min), with access to 50 food pellets (45 mg each, dustless precision pellets, BioServ Flemington, NJ) previously placed inside the food-magazine. Once the animal ate all food-pellets and presented 150 nose-pokes (as measured by a photocell) into the food-magazine, the autoshaping program was initiated.

#### Autoshaping training

The autoshaping program consisted of discrete trials, each composed of the presentation of the retractile and illuminable lever press operandum for 8s (conditioned stimulus, CS) followed by delivery of a 45 mg food pellet (unconditioned stimulus, US) with an inter-trial time (ITT) of 60s. Once the animal pressed the lever in response to the CS, the trial was shortened, the lever was retracted, the light was turned off, and a food pellet (US) was immediately delivered, followed by the ITT. The response during CS was regarded as a conditioned response (CR), and its increase or decrease was considered as an enhancement or impairment measure of memory, respectively [19,20].

### Enzyme-linked immunosorbent assay

After lethal pentobarbital injection, rats were craneotomized and brain samples were rapidly excised. The hippocampus was dissected, weighed, and snap frozen in liquid nitrogen prior to storage at -70°C. After four weeks of freezing, tissue samples were homogenized in ice-cold homogenization buffer consisting of 100mM Tris/HCl (pH 7), 2% bovine serum albumin (BSA), 1M NaCl, 4mM EDTA, 2% Triton X-100, 0.1% NaN_3_, and the following protease inhibitors: 5μg/mL aprotinin, 0.5μg/mL antipain, 157μg/mL benzamidine, 0.1μg/mL pepstatin A, and 17μg/mL phenylmethyl-sulphonyl fluoride. Homogenates were prepared in approximately 20 volumes of the homogenization buffer to tissue-wet weight and centrifuged at 14,000g for 30 minutes. The resulting supernatants were used for the BDNF assay. Samples were analyzed by triplicate, following the instructions of the ChemiKine^™^ BDNF Sandwich ELISA Kit (Millipore, USA). Absorbance was measured in a microplate spectrophotometer at a wavelength of 450nm (MultiSkan, Thermo Scientific, Finland).

### Statistical methods

Statistical analysis was performed using Prism 6 software (Prism 6.0, GraphPad Software Inc., San Diego, CA, USA). Data is expressed as mean ± standard deviation of the mean (SD) to better represent different sample sizes among experiments. All data sets were analyzed for normality using the D’Agostino & Pearson omnibus K2 normality test. MWM evaluations were analyzed using two-factor ANOVA for repeated measures. Student’s T-test was used to analyze autoshaping evaluations and BDNF concentrations. In order to calculate the size of the effect on autoshaping evaluations, we used Cohen’s d test, an effect size test used to indicate the standardized difference between two means. This test can be used alongside T-test and ANOVA results [21]. P-values less than or equal to 0.05 were considered statistically significant.

## Results

### Cop-1 enhances spatial learning memory in adult rats

**Fig 1:**
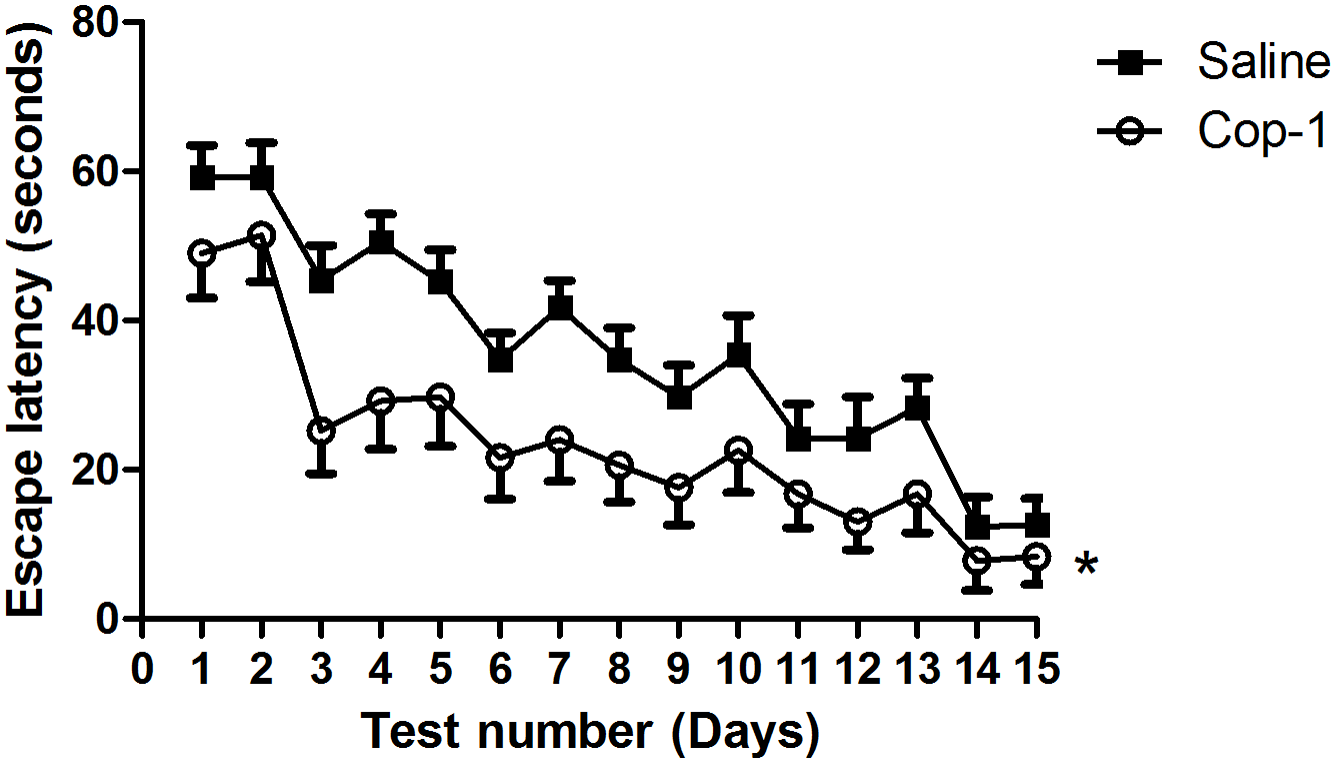
Effect of Cop-1 immunization on cognitive improvement in adult rats. Adult rats (6 months old) were immunized and evaluated using the MWM test. Cop-1 immunization was capable of improving spatial memory at these stages. Each point represents mean ± SD of 8 rats. *p<0.0001, Two-factor ANOVA for repeated measures. This experiment is one of three in which the same effect was observed.

In the first experiment, we explored the effect of Cop-1 on the spatial learning memory of adult rats. For this purpose, sixteen 6-month-old rats were injected either with saline solution (saline, n = 8) or Cop-1 (Cop-1, n = 8). All rats were evaluated daily for 15 days using the MWM behavioral test. Fig 1 shows that Cop-1-treated rats presented significantly shorter latency swim times than saline-treated ones (p< 0.0001; F = 83.23, Two-factor ANOVA for repeated measures). Initially, Cop-1-treated animals presented a shorter latency time (49 ± 3s) when compared to those treated with only saline solution (59 ± 2s). This difference increased after the third day of evaluation, with rats in the experimental group showing a latency time of 25 ± 2 seconds, compared to the latency time of 45 ± 3 seconds for the control group.

### Cop-1 improves spatial learning memory at an advanced age when administered in the early stages of life

**Fig 2:**
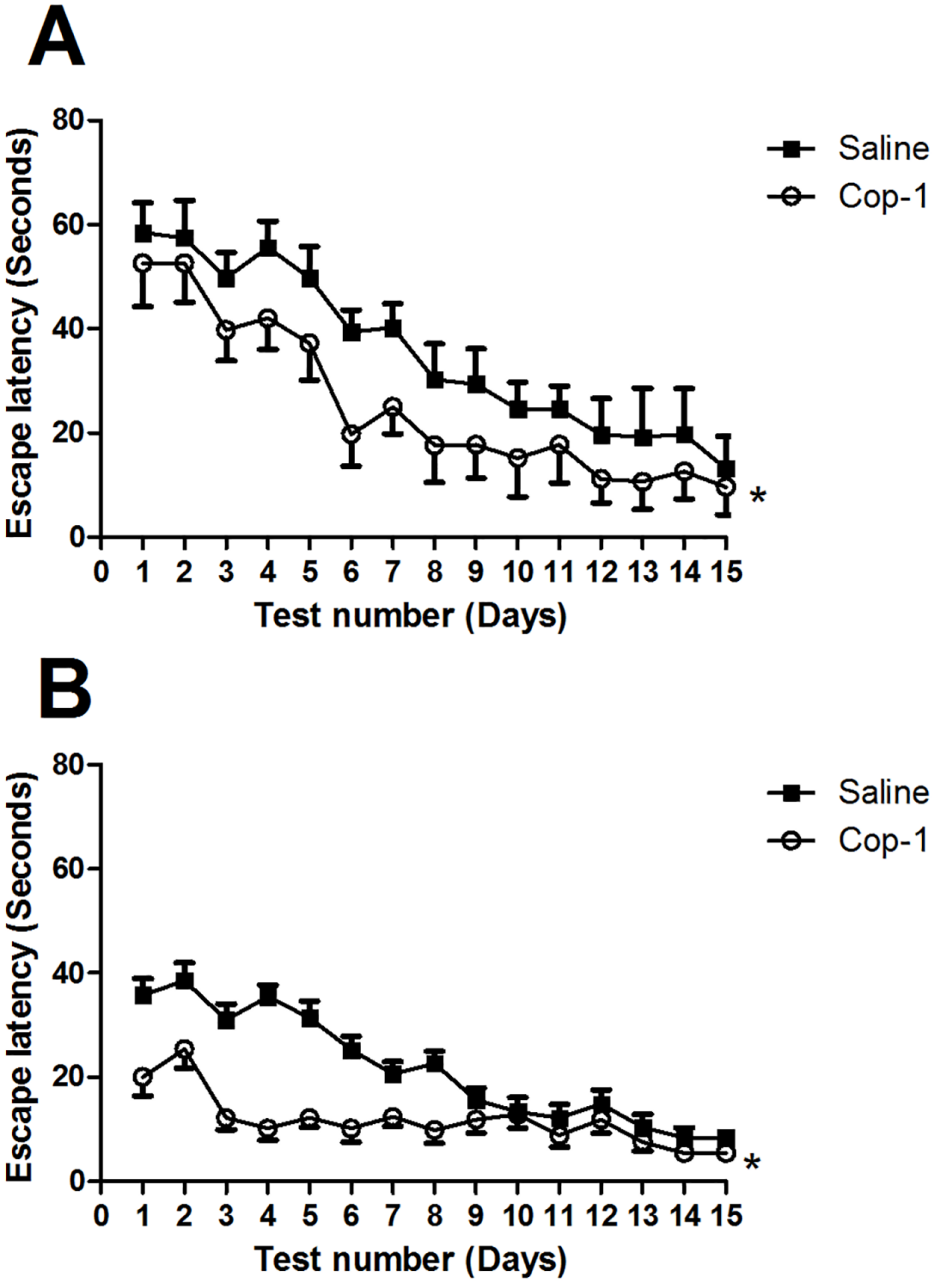
Effect of Cop-1 immunization on cognitive improvement of adult rats when administered from the early stages of life. Young animals (3 months old) were immunized and evaluated at 3 (A) and 6 months old (B) using the MWM test. Immunization with Cop-1 improved cognition at early and later stages of life. Each point represents mean ± SD of 8 rats. *p< 0.0001, Two-factor ANOVA for repeated measures. This experiment is one of three in which the same effect was observed.

In order to elucidate whether Cop-1 is capable of inducing a preventive effect on CI, a second experiment was conducted, in which, either Cop-1 (n = 8) or saline solution (n = 8) were administered to 3-month-old rats. Spatial learning memory was evaluated with the MWM test 15 days and 3 months after administration. Evaluations performed 15 days after treatment demonstrated that Cop-1 administration induced a significant improvement (p< 0.0001, F = 89.14; Two factor ANOVA for repeated measures) in memory when compared to that observed in control rats (Fig 2A). Three months later, animals treated with Cop-1 maintained the improvement in memory (p< 0.0001, F = 19.86; Two factor ANOVA for repeated measures). From initial evaluations, the latency time presented by Cop-1-treated rats was almost half (20.0 ± 3.5s) of the time presented by saline-treated animals (35.8 ± 3s) (Fig 2B). The difference observed between these groups was higher on day 4 of evaluation (Cop-1: 10.20 ± 2.2s, Saline: 35.40 ± 2.3s).

### The effect of Cop-1 on learning memory analyzed by the Pavlovian autoshaping test

**Fig 3:**
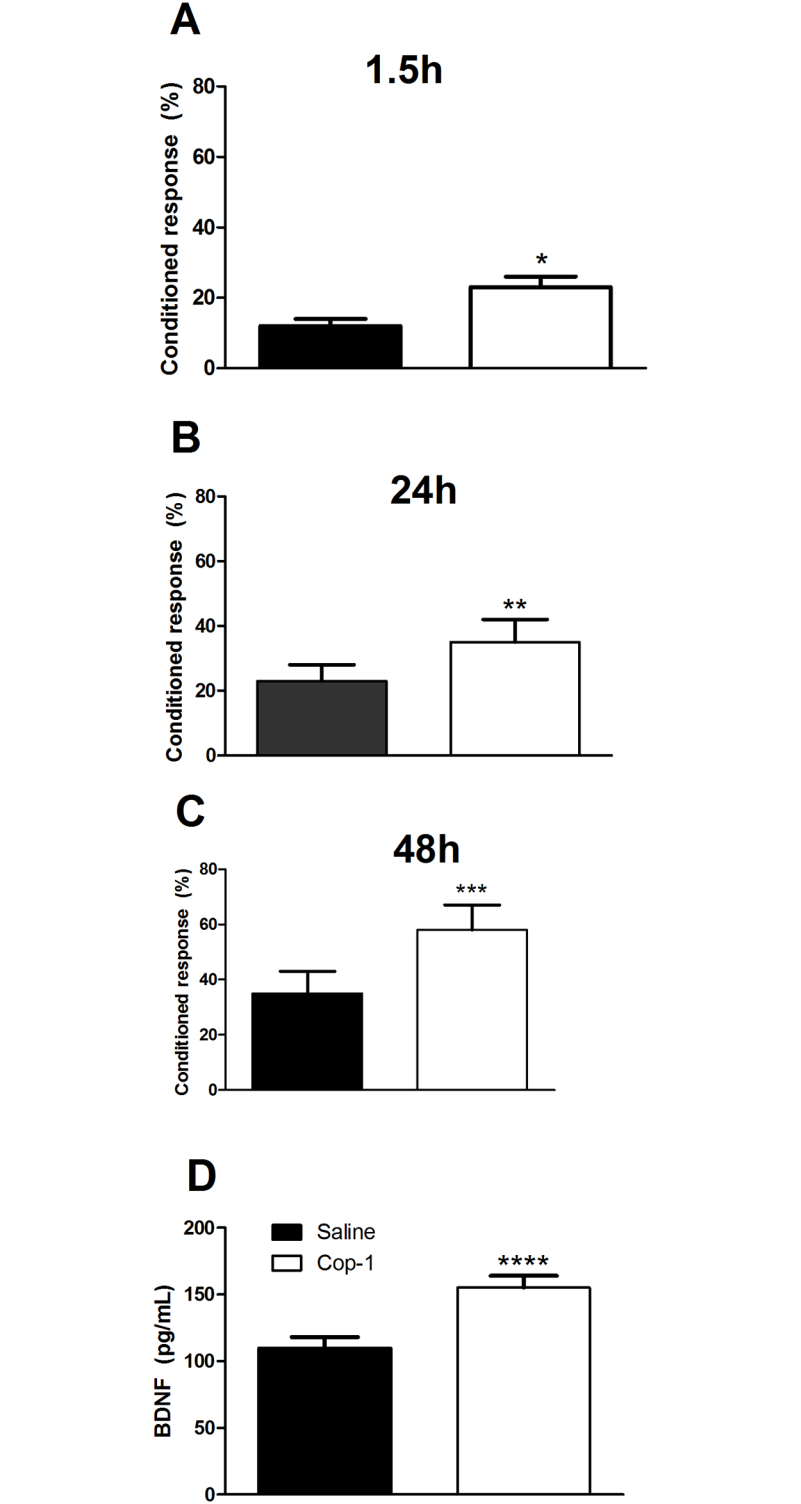
Cop-1 immunization improved associative memory and increased BDNF levels in adult rats. Adult animals (6 months old) were immunized and evaluated using the autoshaping test. Cop-1 immunization was capable of improving associative memory at 1.5 (A), 24 (B), and 48 (C) h after training. Likewise, Cop-1 induced a significant increase of BDNF at the hippocampus (D). Each bar represents mean ± SD of 8 (Figures A, B and C) or 4 (Figure D) rats. *p < 0.03, ** p = 0.01, *** p = 0.05; **** p < 0.01; Student’s T test. This experiment is one of two in which the same effect was observed.

In a Pavlovian autoshaping test, designed to evaluate associative memory, a food pellet is delivered immediately after pressing a retractable lever, acting as a conditioned stimulus [22]. In order to support the findings of our previous experiment, we performed the autoshaping test in adult rats in two additional experiments. In the first, 6–month-old rats were treated either with saline (n = 8) or Cop-1 + saline solution (n = 8). Fig 3 shows that animals treated with Cop-1 presented a higher percentage of conditioned responses; this was observed at 1.5 (Fig 3A, p = 0.03, F = 1.7; Student’s T-test), 24 (Fig 3B, p = 0.01, F = 1.1; Student’s T-test), and 48 hours (Fig 3C, p = 0.05, F = 1.2; Student’s T-test) after the training session. In order to strengthen these results, the data was analyzed with Cohen’s d test. Results from this test evidenced a significant impact of Cop-1 on learning memory at 1.5 (Cohen’s d = 2.0, r = 0.70), 24 (Cohen’s d = 1.15, r = 0.50), and 48 hours (Cohen’s d = 1.81, r = 0.67) after the training session. In the same groups of rats, we evaluated the concentration of BDNF, a molecule produced by Cop-1 activated-lymphocytes [23] and strongly related to cognitive function [24]. BDNF concentrations were analyzed in the hippocampal area of 4 randomly selected rats. Results show a significant increase of BDNF concentrations in animals treated with Cop-1 (155± 9 pg/mL) when compared to those receiving only saline solution (110 ± 8 pg/mL, Fig 3D, p < 0.01, F = 1.3; Student’s T test).

**Fig 4:**
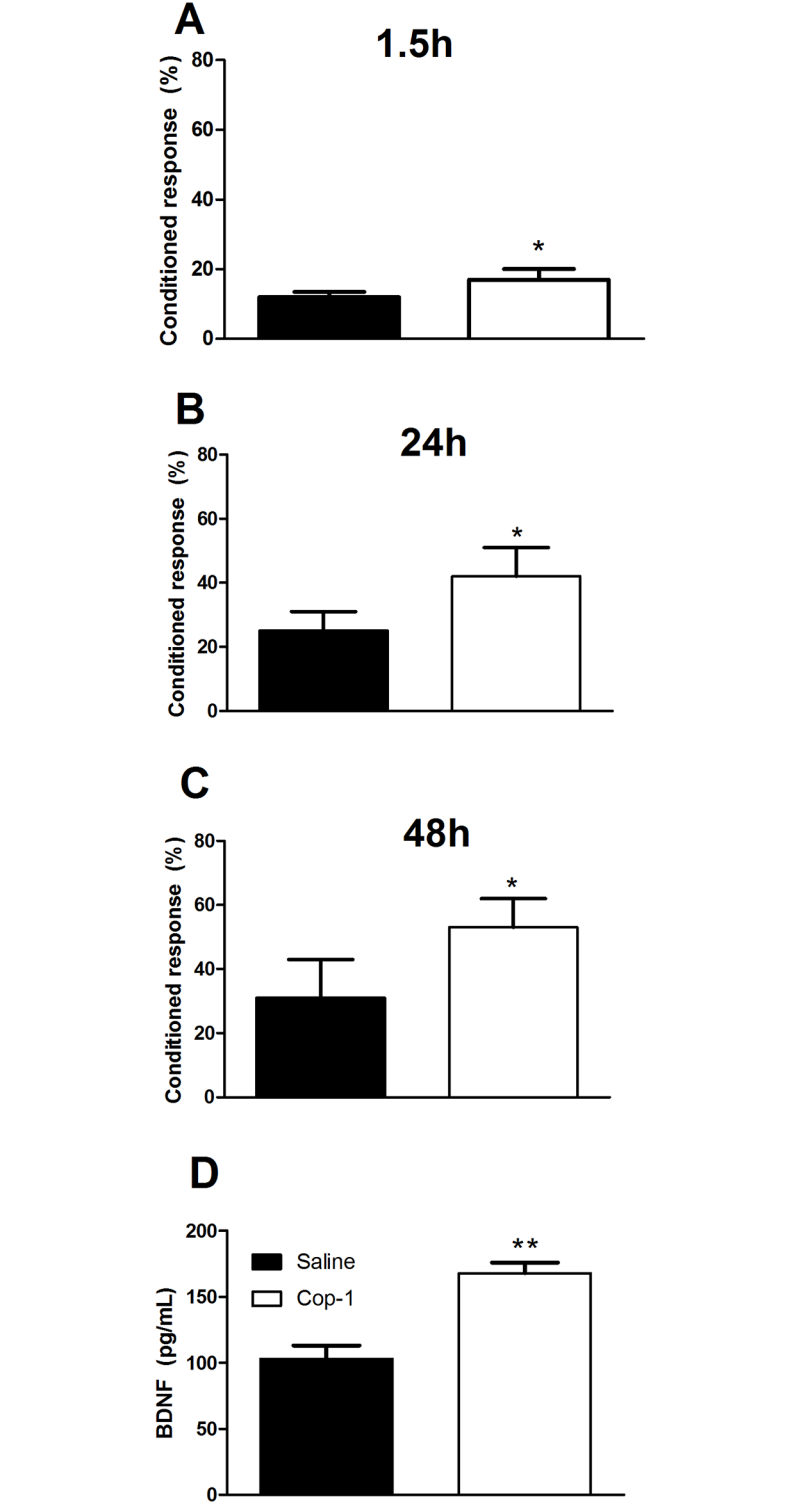
Cop-1 immunization improved associative memory and increased BDNF levels in adult rats, when administered from the early stages of life. Young animals (3 months old) were immunized and evaluated at 6 months of age using the autoshaping test. Cop-1 immunization was capable of improving associative memory at 1.5 (A), 24 (B), and 48 (C) hours after training. BDNF levels were also significantly increased (D). Each bar represents mean ± SD of 8 (Figures A, B and C) or 4 (Figure D) rats. *p = 0.05, ** p< 0.01 Student’s T test. This experiment is one of two in which the same effect was observed.

Finally, in the second experiment, 3-month-old rats were inoculated with Cop-1 (n = 8) or saline solution (n = 8). The rats were evaluated three months later, at 6 months of age. Fig 4 shows an improvement in memory in rats receiving Cop-1 therapy. These rats similarly presented a higher percentage of conditioned responses at 1.5 (Fig 4A, p = 0.05, F = 4.0; Student’s T-test), 24 (Fig 4B, p = 0.05, F = 2.1; Student’s T-test) and 48 hours (Fig 4C, p = 0.05, F = 1.2; Student’s T-test) after the training session. The results of Cohen’s d-test showed an important effect of Cop-1 on learning memory for the three evaluated intervals (1.5h: Cohen’s d = 0.8, r = 0.37; 24h: Cohen’s d = 2.0, r = 0.70; 48h: Cohen’s d = 1.8, r = 0.66). Hippocampal BDNF concentrations were significantly higher in Cop-1-treated rats (168 ± 8 pg/mL, mean ± SD) than those observed in saline-treated rats (103 ± 10 pg/mL; Fig 4D, p < 0.01, F = 1.2; Student’s T-test).

## Discussion

CI has been found to result from diverse factors, the pathophysiological mechanisms behind it have been associated to a reduction of BDNF levels and/or a deficient immune function [8,25]. Cognition can be evaluated by assessing memory and learning, two important processes that are significantly diminished in individuals with CI. Memory and learning are mental abilities that are stored in synapses of the brain [26]. Therefore, as the basic cellular mechanism involved in these processes, synaptic plasticity has the ability to improve cognition. Long-term potentiation (LTP) in the hippocampus is perhaps the best studied form of synaptic plasticity, leading to its consideration as the experimental analog of memory [25]. LTP is divided into two phases: a) induction of LTP or early phase LTP (E-LTP) and b) maintenance of LTP, or late phase LTP (L-LTP). According to previous studies, a deficit in the induction and maintenance of LTP correlates with a deficit in memory and learning [27]. BDNF is a prominent regulator of synaptic plasticity and memory formation, and is therefore critical for learning-related synaptic plasticity and the maintenance of long-term memory. Several studies have demonstrated that BDNF facilitates the induction of E-LTP, as well as the induction and maintenance of L-LTP [28,29]. Therefore, BDNF plays a critical role in cognitive function. Previous investigations performed in aged individuals have shown that the *bdnf* gene is down-regulated during later stages of life, leading to a significant reduction in BDNF levels at an advanced age and thus leading to the concomitant CI [25,30]. Based on these results, the restoration of BDNF levels can be proposed as an effective therapeutic strategy for preventing or ameliorating CI. Likewise, a therapy that envisions the stimulation and/or renewal of immune activity in the brain could also positively impact cognitive function.

The present work offers evidence about a practical and effective strategy to increase BDNF concentrations and stimulate or renew immune activity in the CNS. By utilizing anti-Cop-1 lymphocytes’ ability to produce BDNF, we can compensate the decrease in BDNF levels found in the advanced stages of life. Additionally, Cop-1 immunization could be activating and inducing the migration [31] and accumulation [32] of these cells in the CNS, where they interact with resident cells [33]. Therefore, this strategy could restore both immune function and BDNF levels in the CNS.

Our results show that Cop-1 immunization was capable of improving the levels of BDNF in the hippocampus; moreover, it induced a significant improvement in cognitive function in both animals injected at adult stages of life and those immunized from an early stage. The main mechanism through which Cop-1 improves learning/memory appears to be the induction of BDNF expression. In this regard, previous studies have shown that the increased levels of BDNF induced by Cop-1 are strongly associated with improvement in learning/memory in models of chronic cerebral hypoperfusion [14]. Accordingly, our results show that Cop-1 immunization induced a significant increase in BDNF and improved learning/memory in adult rats.

Other possible mechanisms should be considered as promoters of the cognition improvement observed in this study. For instance, the recruitment of immune cells by itself could also be an important factor contributing to improved cognition. Studies have demonstrated that normal neuroimmune interactions are necessary in order to promote learning, memory, and neuroplasticity [34]. Furthermore, neurogenesis could also be participating in cognitive improvement after Cop-1 immunization. With this regard, previous investigations have demonstrated that Cop-1 is capable of inducing neurogenesis [35], which could thereby modify cognitive function [12].

Finally, it is worth mentioning that in this study we evaluated 2 different kinds of memory: spatial (MWM) and associative (autoshaping). In both cases, rats treated with Cop-1 showed a significant improvement. This could be of relevant interest for clinicians, with the recovery of both types of memory indicating an important advance in the treatment of CI. Moreover, the practical strategy proposed by this study allows for an easy translation of this therapy in humans, due to Cop-1’s status as an FDA-approved treatment for MS and commercial availability as Glatiramer Acetate or Copaxone^®^.

The present investigation reports the first instance of Cop-1 treatment enhancing cognitive function in normal young adult rats, suggesting that Cop-1 may be a practical therapeutic strategy potentially useful for treating CI. Further studies utilizing this therapy at later stages of life, such as in middle-aged or aged animals, or in models of degenerative diseases (i.e. spinal cord injury, traumatic brain injury, stroke) where the appearance of CI has also been demonstrated are warranted, and may yield further promising results [36,37].

## Supporting information

S1 Table (XLS)

S2 Table (XLS)

S3 Table (XLS)

S4 Table (XLS)

## References

[1] Nandrajog P, Idris Z, Azlen WN, Liyana A, Abdullah JM. The use of event-related potential (P300) and neuropsychological testing to evaluate cognitive impairment in mild traumatic brain injury patients. Asian J Neurosurg. 2016;0: 0 doi: 10.4103/1793-5482.180921 28761523 10.4103/1793-5482.180921 PMC5532930

[2] Wu J, Zhao Z, Sabirzhanov B, Stoica BA, Kumar A, Luo T, et al Spinal cord injury causes brain inflammation associated with cognitive and affective changes: role of cell cycle pathways. J Neurosci. 2014;34: 10989–11006. doi: 10.1523/JNEUROSCI.5110-13.2014 25122899 10.1523/JNEUROSCI.5110-13.2014 PMC4131014

[3] Halai AD, Woollams AM, Lambon Ralph MA. Triangulation of language-cognitive impairments, naming errors and their neural bases post-stroke. NeuroImage Clin. Elsevier; 2018;17: 465–473. doi: 10.1016/j.nicl.2017.10.037 29159059 10.1016/j.nicl.2017.10.037 PMC5683039

[4] Ritchie K. Mild cognitive impairment: An epidemiological perspective. Dialogues in Clinical Neuroscience. 2004 pp. 401–408. 22034212 10.31887/DCNS.2004.6.4/kritchie PMC3181815

[5] Roberts R, Knopman DS. Classification and Epidemiology of MCI. Clin Geriatr Med. 2013;29: 753–772. doi: 10.1016/j.cger.2013.07.003 24094295 10.1016/j.cger.2013.07.003 PMC3821397

[6] Ritchie K, Leibovici D, Ledésert B, Touchon J. A typology of sub-clinical senescent cognitive disorder. Br J Psychiatry. 1996;168: 470–476. doi: 10.1192/bjp.168.4.470 8730944 10.1192/bjp.168.4.470

[7] Brynskikh A, Warren T, Zhu J, Kipnis J. Adaptive immunity affects learning behavior in mice. Brain Behav Immun. 2008;22: 861–869. doi: 10.1016/j.bbi.2007.12.008 18249087 10.1016/j.bbi.2007.12.008

[8] Kipnis J, Cohen H, Cardon M, Ziv Y, Schwartz M. T cell deficiency leads to cognitive dysfunction: Implications for therapeutic vaccination for schizophrenia and other psychiatric conditions. Proc Natl Acad Sci. 2004;101: 8180–8185. doi: 10.1073/pnas.0402268101 15141078 10.1073/pnas.0402268101 PMC419577

[9] Blanco Y, Moral EA, Costa M, Gómez-Choco M, Torres-Peraza JF, Alonso-Magdalena L, et al Effect of glatiramer acetate (Copaxone®) on the immunophenotypic and cytokine profile and BDNF production in multiple sclerosis: A longitudinal study. Neurosci Lett. 2006;406: 270–275. doi: 10.1016/j.neulet.2006.07.043 16934924 10.1016/j.neulet.2006.07.043

[10] Aharoni R. Immunomodulation neuroprotection and remyelination—The fundamental therapeutic effects of glatiramer acetate: A critical review [Internet]. Journal of Autoimmunity. 2014 pp. 81–92. doi: 10.1016/j.jaut.2014.05.005 24934599 10.1016/j.jaut.2014.05.005

[11] Shang Y, Wang X, Shang X, Zhang H, Liu Z, Yin T, et al Repetitive transcranial magnetic stimulation effectively facilitates spatial cognition and synaptic plasticity associated with increasing the levels of BDNF and synaptic proteins in Wistar rats. Neurobiol Learn Mem. 2016;134: 369–378. doi: 10.1016/j.nlm.2016.08.016 27555233 10.1016/j.nlm.2016.08.016

[12] Urbach A, Baum E, Braun F, Witte OW. Cortical spreading depolarization increases adult neurogenesis, and alters behavior and hippocampus-dependent memory in mice. J Cereb Blood Flow Metab. SAGE PublicationsSage UK: London, England; 2017;37: 1776–1790. doi: 10.1177/0271678X16643736 27189903 10.1177/0271678X16643736 PMC5435280

[13] Arya A, Gangwar A, Singh SK, Roy M, Das M, Sethy NK, et al Cerium oxide nanoparticles promote neurogenesis and abrogate hypoxia-induced memory impairment through AMPK-PKC-CBP signaling cascade. Int J Nanomedicine. Dove Press; 2016;11: 1159–1173. doi: 10.2147/IJN.S102096 27069362 10.2147/IJN.S102096 PMC4818056

[14] Chen L, Yao Y, Wei C, Sun Y, Ma X, Zhang R, et al T cell immunity to glatiramer acetate ameliorates cognitive deficits induced by chronic cerebral hypoperfusion by modulating the microenvironment. Sci Rep. Nature Publishing Group; 2015;5: 14308 doi: 10.1038/srep14308 26391515 10.1038/srep14308 PMC4585746

[15] Garthe A, Kempermann G. An old test for new neurons: Refining the Morris water maze to study the functional relevance of adult hippocampal neurogenesis. Frontiers in Neuroscience. 2013 doi: 10.3389/fnins.2013.00063 23653589 10.3389/fnins.2013.00063 PMC3642504

[16] Gonzalez R, Chávez-Pascacio K, Meneses A. Role of 5-HT5A receptors in the consolidation of memory. Behav Brain Res. 2013;252: 246–251. doi: 10.1016/j.bbr.2013.05.051 23735322 10.1016/j.bbr.2013.05.051

[17] Meneses A. Frameworking memory and serotonergic markers. Rev Neurosci. 2017;28: 455–497. doi: 10.1515/revneuro-2016-0079 28343185 10.1515/revneuro-2016-0079

[18] Aparicio-Nava L, Márquez-García LA, Meneses A. Effects of 5-HT5A receptor blockade on amnesia or forgetting. Behav Brain Res. 2018; https://doi.org/10.1016/j.bbr.2018.01.009 10.1016/j.bbr.2018.01.009 29330003

[19] Meneses A. A Pharmacological Analysis of an Associative Learning Task: 5-HT(1) to 5-HT(7) Receptor Subtypes Function on a Pavlovian/Instrumental Autoshaped Memory. Learn Mem. Cold Spring Harbor Laboratory Press; 2003;10: 363–372. doi: 10.1101/lm.60503 14557609 10.1101/lm.60503 PMC218002

[20] Meneses A, Perez-Garcia G, Liy-Salmeron G, Ponce-López T, Lacivita E, Leopoldo M. 5-HT7 receptor activation: Procognitive and antiamnesic effects. Psychopharmacology (Berl). 2015;232: 595–603. doi: 10.1007/s00213-014-3693-0 25074446 10.1007/s00213-014-3693-0

[21] Rosenthal R. Parametric Measures Of Effect Size Handbook of Research Synthesis, The. Russell Sage Foundation; 1994 pp. 231–244. http://www.jstor.org/stable/10.7758/9781610441377.20

[22] Srey CS, Maddux J-MN, Chaudhri N. The attribution of incentive salience to Pavlovian alcohol cues: a shift from goal-tracking to sign-tracking. Front Behav Neurosci. Frontiers; 2015;9: 54 doi: 10.3389/fnbeh.2015.00054 25784867 10.3389/fnbeh.2015.00054 PMC4347508

[23] Azoulay D, Vachapova V, Shihman B, Miler A, Karni A. Lower brain-derived neurotrophic factor in serum of relapsing remitting MS: Reversal by glatiramer acetate. J Neuroimmunol. Elsevier; 2005;167: 215–218. doi: 10.1016/j.jneuroim.2005.07.001 16083971 10.1016/j.jneuroim.2005.07.001

[24] McAllister TW, Tyler AL, Flashman LA, Rhodes CH, McDonald BC, Saykin AJ, et al Polymorphisms in the Brain-Derived Neurotrophic Factor Gene Influence Memory and Processing Speed One Month after Brain Injury. J Neurotrauma. Mary Ann Liebert, Inc. 140 Huguenot Street, 3rd Floor New Rochelle, NY 10801 USA; 2012;29: 1111–1118. doi: 10.1089/neu.2011.1930 22188054 10.1089/neu.2011.1930 PMC3325555

[25] Penner MR, Roth TL, Barnes CA, Sweatt JD. An epigenetic hypothesis of aging-related cognitive dysfunction. Front Aging Neurosci. Frontiers; 2010;2: 9 doi: 10.3389/fnagi.2010.00009 20552047 10.3389/fnagi.2010.00009 PMC2874394

[26] Lu Y, Christian K, Lu B. BDNF: A key regulator for protein synthesis-dependent LTP and long-term memory? Neurobiol Learn Mem. 2008;89: 312–323. doi: 10.1016/j.nlm.2007.08.018 17942328 10.1016/j.nlm.2007.08.018 PMC2387254

[27] Barnes CA. Memory deficits associated with senescence: A neurophysiological and behavioral study in the rat. J Comp Physiol Psychol. 1979;93: 74–104. doi: 10.1037/h0077579 221551 10.1037/h0077579

[28] Yano H, Ninan I, Zhang H, Milner TA, Arancio O, Chao M V. BDNF-mediated neurotransmission relies upon a myosin VI motor complex. Nat Neurosci. Nature Publishing Group; 2006;9: 1009–1018. doi: 10.1038/nn1730 16819522 10.1038/nn1730

[29] Pang PT, Lu B. Regulation of late-phase LTP and long-term memory in normal and aging hippocampus: Role of secreted proteins tPA and BDNF [Internet]. Ageing Research Reviews. 2004 pp. 407–430. doi: 10.1016/j.arr.2004.07.002 15541709 10.1016/j.arr.2004.07.002

[30] Hattiangady B, Rao MS, Shetty GA, Shetty AK. Brain-derived neurotrophic factor, phosphorylated cyclic AMP response element binding protein and neuropeptide Y decline as early as middle age in the dentate gyrus and CA1 and CA3 subfields of the hippocampus. Exp Neurol. 2005;195: 353–371. doi: 10.1016/j.expneurol.2005.05.014 16002067 10.1016/j.expneurol.2005.05.014

[31] Prat A, Biernacki K, Antel JP. Th1 and Th2 lymphocyte migration across the human BBB is specifically regulated by interferon β and copolymer-1. J Autoimmun. 2005;24: 119–124. doi: 10.1016/j.jaut.2005.01.004 15829404 10.1016/j.jaut.2005.01.004

[32] Aharoni R, Meshorer A, Sela M, Arnon R. Oral treatment of mice with copolymer 1 (glatiramer acetate) results in the accumulation of specific Th2 cells in the central nervous system. J Neuroimmunol. 2002;126: 58–68. doi: 10.1016/S0165-5728(02)00053-X 12020957 10.1016/s0165-5728(02)00053-x

[33] Qian S, Tang Y, Cheng L, Sun X, Tian J, Zhou C. Interaction of copolymer-1-activated T cells and microglia in retinal ganglion cell protection. Clin Exp Ophthalmol. 2013;41: 881–890. doi: 10.1111/ceo.12110 23566072 10.1111/ceo.12110

[34] Di Benedetto S, Müller L, Wenger E, Düzel S, Pawelec G. Contribution of neuroinflammation and immunity to brain aging and the mitigating effects of physical and cognitive interventions. Neurosci Biobehav Rev. 2017;75: 114–128. doi: 10.1016/j.neubiorev.2017.01.044 28161508 10.1016/j.neubiorev.2017.01.044

[35] Cruz Y, Lorea J, Mestre H, Kim-Lee JH, Herrera J, Mellado R, et al Copolymer-1 promotes neurogenesis and improves functional recovery after acute ischemic stroke in rats. Boltze J, editor. PLoS One. Public Library of Science; 2015;10: e0121854 doi: 10.1371/journal.pone.0121854 25821957 10.1371/journal.pone.0121854 PMC4378896

[36] Craig A, Guest R, Tran Y, Middleton J. Cognitive Impairment and Mood States after Spinal Cord Injury. J Neurotrauma. 2017;34: 1156–1163. doi: 10.1089/neu.2016.4632 27717295 10.1089/neu.2016.4632

[37] Chander RJ, Lam BYK, Lin X, Ng AYT, Wong APL, Mok VCT, et al Development and validation of a risk score (CHANGE) for cognitive impairment after ischemic stroke. Sci Rep. Springer US; 2017;7: 12441 doi: 10.1038/s41598-017-12755-z 28963553 10.1038/s41598-017-12755-z PMC5622067

